# Editorial: Sex differences in renal physiology

**DOI:** 10.3389/fphys.2023.1332289

**Published:** 2023-11-20

**Authors:** Purnima Singh, Shubha Ranjan Dutta, Jennifer C. Sullivan

**Affiliations:** ^1^ Department of Pharmacology, Addiction Sciences and Toxicology, University of Tennessee Health Science Center, Memphis, TN, United States; ^2^ Department of Physiology, Augusta University, Augusta, GA, United States

**Keywords:** sex-difference, renal function, computational, RAGE, fructose, acute kidney injury, HELLP syndrome

The major functions of kidneys are the regulation of blood pressure, excretion of metabolic wastes, and regulation of electrolytes and acid-base balance. There are well-established sex differences in renal function across many species, including humans and rodents. In fact, many studies have suggested the female sex as a protective factor in the progression of many renal diseases, both clinically and in experimental animal models. However, the influence of biological sex on renal disease development and progression remains poorly understood. As such, there continues to be a high degree of interest in better understanding the mechanisms controlling renal function under both physiological and pathophysiological conditions in both sexes. To that end, *Frontiers in Physiology-Renal Physiology* launched a recent Call for Papers on *Sex differences in renal physiology*. Regarding this Research Topic, computational models of sex differences in epithelial water and solute transport along the nephrons and identification of risk and prognosis factors associated with acute kidney injury (AKI) in pregnant patients are very attractive and promising.

Stadt and Layton developed the first sex-specific computational models of epithelial water and solute transport along the nephrons from male and female mouse kidneys. These models can serve as a valuable tool for the analysis of findings related to genetic modification from experiments conducted in rats and mice. These models can be useful for the interpretation of the differences in the abundance of apical and basolateral transporters, glomerular filtration rate, and tubular dimensions between the sexes. Using their computational models, the authors showed that the mouse kidney proximal tubules reabsorb less Na + than rats, with Na + reabsorption higher in males than females in both species. They also proposed that the females exhibit enhanced water and Na + transport along distal nephrons in both species due to the abundance of key Na + transporters resulting in similar urine excretion between the sexes. Therefore, this model can be applicable to all mammalian kidneys, irrespective of species to enhance the understanding of physiological and pathological mechanisms for the treatment of diseases without introducing intended genetic manipulation from compensation.

Monteiro et al. reported metabolic and renal changes due to fructose overload introduced after weaning in rats, with greater effects in males than females. The authors showed that fructose intake was associated with increased blood pressure, body weight, plasma triglycerides, renal macrophage infiltration, decreased sodium, and potassium excretion, and decreased renal eNOS expression in both male and female rats. However, the excretion of sodium, potassium, and magnesium was higher in females. They further showed that males receiving fructose had a reduced glomerular filtration rate compared to females. These observations indicate that nutritional status in the early developmental stage is crucial in an individual’s health and improper dietary changes in early life could become a cause of non-infectious diseases in the future, particularly in males.

Bajwa et al. examined the role of the receptors for advanced glycation end products (RAGE) that interact with multiple pro-inflammatory/pro-oxidative ligands and are considered to have potential pathophysiological effects on the kidneys. They reported that the RAGE mRNA expression in the kidney cortex of old (>70 weeks) wild-type female mice was higher than that in males of similar age. The expression for macrophage scavenger receptors (MSR) that mediates the endocytic uptake of AGEs *in vivo* in the kidneys also increased in aged mice, irrespective of sex and genotype (wild-type and RAGE knockout), and this increase in MSR correlated with the increase in AGE content. Interestingly, there was an age-dependent increase in renal damage (NGAL and KIM1) associated with fibrosis, hyaline casts deposition, and accumulation of immune infiltrates comprising mostly of B220-positive B cells, and some B-1a (CD19^+^ CD43^+^ CD5^+^) in RAGE knockout (KO) mice, irrespective of sex. However, there was an age-based increase in pro-fibrotic and pro-inflammatory markers (IL-6, TNF, TGF-β1, and SNAIL1) in RAGE-KO male mice. These observations suggest that the male sex predisposes towards premature aging-dependent renal damage irrespective of RAGE.

Wang et al. identified the risk and prognosis factors associated with acute kidney injury (AKI) in pregnant patients with hemolysis, elevated liver enzymes, and low platelet count (HELLP) syndrome. Using data from 110 pregnant HELLP patients with or without AKI, they reported the lowest hemoglobin and highest bleeding incidence as independent risk factors for AKI onset and infection as independent risk factors for maternal mortality. Serum creatinine was reported to be the independent risk factor for both AKI onset and maternal mortality. Moreover, these were different based on the severity of AKI. Thus, the findings of this study support the present view of the diagnosis and treatment of HELLP and AKI patients.

The goal of this Research Topic was to identify sex-specific differences in renal function to promote further understanding of physiological differences between male and female kidneys in animal models and in humans, to improve current therapies as well as provide opportunities for the development of novel protective strategies in nephrology. This Research Topic was also designed to stimulate thought and interest among the renal research community as they consider sex as a biological variable in future research projects.

